# Physical Activity Assessment in Patients With Nephrotic Syndrome and the Impact of COVID-19: A Study Using the International Physical Activity Questionnaire and Accelerometry

**DOI:** 10.7759/cureus.82126

**Published:** 2025-04-12

**Authors:** Hiroki Nishiwaki, Sho Sasaki, Daisuke Ichikawa, Yugo Shibagaki, Takeshi Hasegawa, Fumihiko Koiwa

**Affiliations:** 1 Division of Nephrology, Department of Internal Medicine, Showa Medical University Fujigaoka Hospital, Yokohama, JPN; 2 Institute of Clinical Epidemiology (iCE), Showa Medical University, Tokyo, JPN; 3 Department of Nephrology, Iizuka Hospital, Iizuka, JPN; 4 Section of Education for Clinical Research, Kyoto University Hospital, Kyoto, JPN; 5 Division of Nephrology and Hypertension, Department of Internal Medicine, St. Marianna University School of Medicine, Kawasaki, JPN; 6 Department of Hygiene, Public Health, and Preventive Medicine, Graduate School of Medicine, Showa Medical University, Tokyo, JPN; 7 Department of Nephrology, Graduate School of Medicine, Showa Medical University, Tokyo, JPN; 8 Showa University Research Administration Center (SURAC), Showa Medical University, Tokyo, JPN

**Keywords:** accelerometer, international physical activity questionnaire, kidney function, nephrotic syndrome, physical activity, validation

## Abstract

Background

Nephrotic syndrome (NS) typically involves proteinuria, reduced levels of albumin in the blood, and swelling. It may also lead to complications, including an increased risk of thrombosis and a decline in kidney function. While pharmacological treatments are primary, physical activity (PA) may play a role in improving outcomes. However, limited data exist on PA levels in patients with NS, particularly regarding the validity of self-reported PA assessments. The COVID-19 pandemic also introduced significant lifestyle changes, potentially impacting PA and kidney function. This study aimed to (1) compare self-reported PA using the International Physical Activity Questionnaire (IPAQ) with objectively measured PA via accelerometry in patients with NS and (2) evaluate changes in PA and kidney function before and during the COVID-19 pandemic.

Methods

This multicenter cohort study recruited outpatients aged ≥15 years with primary NS between 2019 and 2020. Participants completed IPAQ and wore accelerometers for at least seven days. PA intensities were categorized as light PA (LPA), moderate-to-vigorous PA (MVPA), or sedentary behavior (SB). The association between IPAQ and accelerometers by activity measurement method describes the values measured for each activity intensity and shows the correlation coefficient. Kidney function was assessed using estimated glomerular filtration rate (eGFR) and urinary protein-to-creatinine ratio (UPCR). PA and kidney function were compared before (December 2019 to March 2020) and during (June to December 2020) the pandemic.

Results

Seventeen participants were included in the IPAQ-accelerometer comparison, and eight were analyzed for pre-pandemic versus pandemic PA changes. Across all intensity levels, IPAQ tended to underestimate PA compared to accelerometers, but this was not statistically significant (IPAQ vs. accelerometer in total PA: 56.6 vs. 652.6 min/day, p = 0.56). IPAQ showed no correlation with accelerometer-measured LPA but demonstrated a moderate correlation with MVPA. During the pandemic, MVPA decreased by 0.88% (95% CI: -5.08 to 3.33%), while SB increased by 0.59% (95% CI: -10.1% to 11.3%). Step count decreased by 810 steps/day (95% CI: -3,597 to 1,977). Kidney function remained stable, with eGFR decreasing by 4.1 mL/min/1.73 m² (p = 0.98) and UPCR increasing by 1.9 g/gCre (p = 0.17), although these changes were not statistically significant.

Conclusions

IPAQ underestimated PA compared to accelerometry, particularly for low-intensity activity. During the pandemic, PA decreased, while sedentary time increased, although kidney function remained stable. These findings highlight the need for objective PA measurement in patients with NS and suggest that pandemic-related lifestyle changes may have influenced PA behaviors. Further research is warranted to assess the long-term impact of PA on kidney outcomes in this population.

## Introduction

Nephrotic syndrome (NS) is a kidney disorder characterized by heavy proteinuria, hypoalbuminemia, and edema [1]. Patients with NS often experience complications such as dyslipidemia, increased thrombotic risk, and progressive kidney dysfunction. Although pharmacological interventions, including corticosteroids and immunosuppressants, are the primary treatment modalities, nonpharmacological strategies such as lifestyle modifications, including physical activity (PA), may play a role in improving clinical outcomes. However, there is limited evidence on the PA levels of patients with NS and the most appropriate methods for assessing their activity.

Accurate assessment of PA is crucial for understanding its impact on health outcomes. Subjective measures such as the International Physical Activity Questionnaire (IPAQ) are widely used in epidemiological studies due to their ease of administration [2,3]. However, these self-reported tools may be subject to recall bias and social desirability bias, potentially leading to inaccurate estimations of activity levels [4]. In contrast, accelerometers provide objective measurements of PA, offering greater precision in capturing movement patterns and intensity [5,6]. While the IPAQ has been validated in various populations, its accuracy in patients with NS remains unclear [7-9]. A direct comparison between IPAQ and accelerometer-derived PA measures in this patient group has yet to be explored.

Additionally, the COVID-19 pandemic led to significant disruptions in daily life, including changes in PA behaviors [10]. Lockdown measures and social restrictions resulted in reduced mobility, increased sedentary time, and altered health behaviors across different populations. Patients with NS, who may already have limitations in PA due to their disease burden, could have been particularly affected. The effects of exercise and PA on proteinuria and kidney function in patients with NS remain unclear. Exercise is known to transiently increase glomerular permeability, potentially leading to elevated urinary protein excretion [11]. As a result, some patients with kidney disease may adopt a pattern of avoiding exercise [12]. The COVID-19 pandemic, which led to an unanticipated reduction in PA, served as a natural experiment to explore the potential impact of decreased activity on these outcomes.

To address these gaps, this study aimed to (1) compare self-reported PA using IPAQ with accelerometer-measured activity levels in patients with primary NS to evaluate the reliability of self-reported measures in patients with NS and (2) examine changes in PA and kidney function before and during the COVID-19 pandemic. By clarifying the accuracy of PA assessment methods and identifying the effects of pandemic-related lifestyle changes, this study provides valuable insights into the management of PA in patients with NS.

## Materials and methods

Study design and setting

This study was planned as a multicenter cohort initiated in 2019 to recruit patients with primary NS and examine the association between accelerometer-measured activity and kidney outcomes in these patients. During the recruitment process, the COVID-19 pandemic emerged, prompting the Japanese government to declare a state of emergency and implement several priority measures to prevent the spread of the virus, starting on April 7, 2020.

In Japan, emergency declarations and priority measures for infectious disease control are issued by the prime minister when the spread of an infectious disease is deemed to have a significant impact on public health and socioeconomic activities [10]. The duration and affected regions are determined by law. Prefectural governors can enforce various restrictions, including recommendations to stay at home, limitations on the use of public facilities, requests for business closures, orders to shorten operating hours for restaurants and other establishments, restrictions on public events, and directives regarding the use of land and buildings for temporary medical facilities or the sale of medical supplies and masks.

Given the potential for unplanned changes in behavioral patterns due to restrictions affecting PA levels in patients with NS, the cohort study was modified. Recruitment of new participants was suspended, and activity levels were reassessed in participants who had previously undergone measurement to evaluate changes before and after the spread of the infection in a single center, Showa University Fujigaoka Hospital. Pre-COVID-19 activity measurements were collected between December 2019 and March 2020, while during COVID-19 measurements were obtained from June to December 2020.

Participants completed a questionnaire at an outpatient clinic and wore an accelerometer at home. Only assessments where accelerometer wear was confirmed for at least seven days were included in the analysis. Both the questionnaire and accelerometers were returned via mail.

Participants

This study included outpatients aged 15 years or older with preserved activities of daily living (ADL) who provided informed consent for PA measurement. Eligible patients had a confirmed diagnosis based on kidney biopsy of either membranous nephropathy or focal segmental glomerulosclerosis (FSGS) or were diagnosed with refractory NS. Refractory NS was defined according to clinical guidelines as the failure to achieve complete or partial remission within six months despite various treatments, including mandatory administration of corticosteroids and immunosuppressive agents [1,13]. Patients were excluded if they had secondary NS, severe infections requiring hospitalization, an ADL score of 60 or lower on the Barthel Index, or viral hepatitis. For participants under 20 years of age, informed consent was additionally obtained from a legal guardian. Patients who did not meet the accelerometer-wearing time requirements described below were excluded from the analysis.

Ethical approval

The study was approved by the Showa Medical University Fujigaoka Hospital Institutional Review Board (approval numbers F2018C50 and F2020C29), and all participants provided written informed consent.

Measurements

Accelerometers

Activity was measured using ActiGraph GT9X 3-axis accelerometers (ActiGraph LLC, Pensacola, Florida, USA). Data were processed using ActiLife (v 6.13.5) software. Non-wear time was calculated using the Troiano algorithm with a 60-second minimum wear period. Participants were asked to wear the accelerometer on their hips while awake for at least seven days and for at least eight hours of continuous wear per day, but not in situations that could damage the device (e.g., underwater activities or contact sports). Patients who did not meet this wearing time were excluded from the analysis. The epoch length was set at 60 seconds, and estimated metabolic equivalents of task (METs) were calculated. Waking wear days included eight or more waking hours. The primary PA variable was vector magnitude average counts per minute. Each 60-second epoch was classified as light PA (LPA; 1.6-2.9 METs), moderate-to-vigorous PA (MVPA; ≥3.0 METs), or sedentary behavior (SB; ≤1.5 METs). The percentage of activity time for each intensity, divided by the wearing time, was used. The number of steps was also measured. All variables were treated as continuous.

Self-Reported PA

Participants completed questionnaires on PA using the IPAQ short form. The IPAQ short form was used to assess PA during a typical week. The IPAQ was developed by the International Consensus Group on Physical Activity Measurement, which included representatives from 12 countries and was supported by WHO. It was designed to enable the estimation of PA levels across populations with different national and sociocultural backgrounds [3]. As the IPAQ is available in multiple languages, including Japanese, no translation was required for this study [2]. The short form consists of eight items and is used to estimate the duration of different intensities of PA performed over the past week. It assesses four types of activity: (1) vigorous-intensity activities such as aerobics; (2) moderate-intensity activities such as leisure cycling; (3) walking or light-intensity activities; and (4) time spent sitting. For each activity intensity, participants were asked to report the number of days per week and the average duration per day (in minutes) that they engaged in the activity continuously for at least 10 minutes. To quantify PA levels, LPA, MVPA, and total PA were estimated by multiplying the duration (minutes per day) by the frequency (days per week). Time spent sitting was not included in the analysis. All variables were treated as continuous.

Demographic Data, Physical Findings, Laboratory Data, and Questionnaire

Age, date of birth, sex, height, weight, activity of daily life assessment, lower extremity edema, blood pressure, Charlson index, pathological diagnosis of NS, and treatment details were obtained from physician interviews, physical examinations, and medical records. Laboratory data, including total protein, serum albumin, blood urea nitrogen, serum creatinine, estimated glomerular filtration rate (eGFR), and urinary protein-to-creatinine ratio (UPCR), were collected from outpatient visits on the days patients provided consent to participate in the study. Additional data were collected on the use of sleeping pills, tobacco use, alcohol consumption, occupation, education level, household income, and marital status.

Statistical analyses

Study variables were described overall using the mean and SD for continuous variables and frequencies and proportions for categorical variables. To account for differences in accelerometer wear time across participants, the durations of PA and SB were normalized by calculating their proportions relative to the total wear time. The Wilcoxon test showed mean differences between the measurement tools, and Spearman’s rank-order correlations were used to examine the strength of the relationship between the PA measured by IPAQ and accelerometers. We illustrated forest plots to describe the distribution of PA and SB in 2019 and 2020. To investigate the changes in PA or SB and kidney function from before to during the COVID-19 outbreak, we performed paired t-tests. All statistical analyses were performed using STATA (version 18.0, StataCorp LLC, College Station, Texas, USA), with the alpha level set at 0.05.

## Results

Nineteen patients were initially enrolled in the planned study, and all patients had their activity measured by both accelerometers and IPAQ before the COVID-19 epidemic. One participant was subsequently excluded due to insufficient wear time of the accelerometer. Among the remaining 18 participants, nine had their activity measured again with the accelerometer during the COVID-19 epidemic; however, one of these was also excluded for insufficient wear time. Consequently, 17 participants underwent analysis using the IPAQ and accelerometer data, and among these, eight participants were included in the analysis of PA before and during the COVID-19 period.

Table 1 shows the characteristics of the included participants in the analysis. There were no missing values in the data obtained. All patients had independent ADL. No participant had COVID-19 during the observation period. Of the 17 participants, nine had membranous nephropathy, six had FSGS, and two had relapsing NS. Of the two cases, one participant had minimal change disease, and one had membranoproliferative glomerulonephritis.

**Table 1:** Participant characteristics The variables are presented as mean and SD for continuous variables and frequencies and proportions for categorical variables. eGFR: estimated glomerular filtration rate; FSGS: focal segmental glomerulosclerosis; mPSL: methylprednisolone; NS: nephrotic syndrome; UPCR: urinary protein-to-creatinine ratio

Variable	All (n = 17)		Analysis before and during the COVID-19 (n = 8)	
	Mean, n	SD, %	Mean, n	SD, %
Age (years)	66.4	-10.5	66	-8.8
Sex (n, %)				
Male	11	64.70%	5	62.50%
Female	6	35.30%	3	37.50%
Education (n, %)				
Junior high school or high school	4	23.50%	1	12.50%
College	4	23.50%	4	50.00%
Graduate school	9	52.90%	3	37.50%
Marital status (n, %)				
Single	1	5.90%	0	0.00%
Married	15	88.20%	7	87.50%
Separated or divorced	1	5.90%	1	12.50%
Job type (n, %)				
Technical worker	5	29.40%	2	25.00%
Manager	3	17.60%	0	0.00%
Service worker	4	23.50%	3	37.50%
Unemployed	3	17.60%	3	37.50%
Other	1	5.90%	0	0.00%
Cause of NS (n, %)				
Membranous nephropathy	9	52.90%	7	87.50%
FSGS	6	35.30%	1	12.50%
Other	2	11.80%	0	0.00%
Disease duration (year, SD)	5.5	3.5	6.8	5.3
Height (cm)	164.4	-6.3	164.5	-7.8
Body weight (kg)	63.7	-10.4	66.2	-13.3
Edema (n, %)	8	47.10%	2	25.00%
Total protein (g/dL)	6.4	-1	6	-0.6
Serum albumin (g/dL)	3.5	-0.5	3.3	-0.7
Blood urea nitrogen (mg/dL)	26.8	-12.9	31.2	-15
eGFR (mL/min/1.73 m²)	42.9	-22.7	44.7	-19.3
UPCR (g/gCre)	3.2	-3.8	2.5	2.8
Predonine use				
History of mPSL pulse therapy (n, %)	0	0.00%	0	0%
Largest amount in the past (mg/day)	21.6	-21.7	30	-20.7
Current dosage (mg/day)	6.1	-7.8	9.6	-9.5
Rituximab (n, %)	1	5.90%	1	12.50%
Cyclosporine (n, %)	2	11.80%	1	12.50%
Tacrolimus (n, %)	3	17.60%	3	37.50%

Table 2 shows activity hours per day measured by IPAQ and activity hours measured by accelerometer for each activity intensity. At all activity intensities, IPAQ underestimated compared to the measurements by the accelerometer. IPAQ did not correlate with accelerometers at low-intensity activity, while MVPA showed a moderate correlation.

**Table 2:** PA for both instruments The unit is min/day. * Statistically significant (p < 0.05) IPAQ: International Physical Activity Questionnaire; LPA: light physical activity; MVPA: moderate-to-vigorous physical activity; PA: physical activity

Activity type	Overall (n = 17)		Correlation coefficient
	IPAQ (min/day)	Accelerometer (min/day)	
LPA	42.4 (14.1-127.3)	627.5 (545.3-703.9)	0.0592
MVPA	0 (0-25.7)	11.0 (8.5-30.2)	0.6883*
Total PA	56.6 (22.7-127.3)	652.6 (601.0-766.2)	0.5644

**Figure 1:**
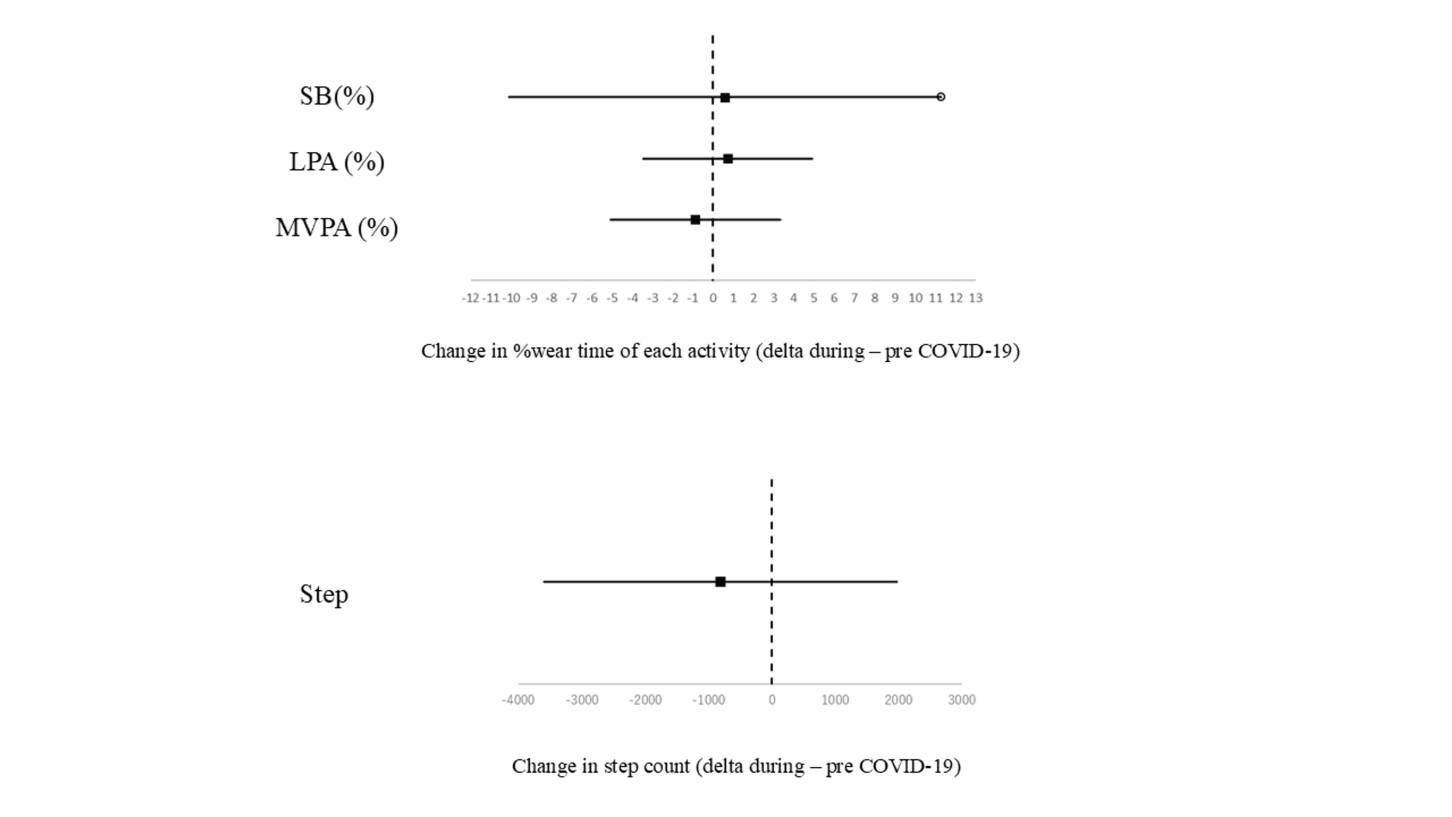
Difference in LPA, MVPA, number of steps, and SB during and before the COVID-19 infection epidemic Dots indicate point estimates, and bars indicate 95% CIs. Each unit represents the percentage of time per wearing time for LPA and MVPA, the difference in steps, and the number of minutes per day for SB. LPA: light physical activity; MVPA: moderate-to-vigorous physical activity; SB: sedentary behavior

Figure 1 shows the difference in activity before and during the COVID-19 epidemic, with a mean change of SB changed +0.59% (95% CI -10.1% to 11.3%), +0.72% (95% CI -3.46% to 4.91%) for LPA and -0.88% (95% CI -5.08% to 3.33%) for MVPA. Steps taken were -810 steps (95% CI -3,597, +1,977).

**Figure 2:**
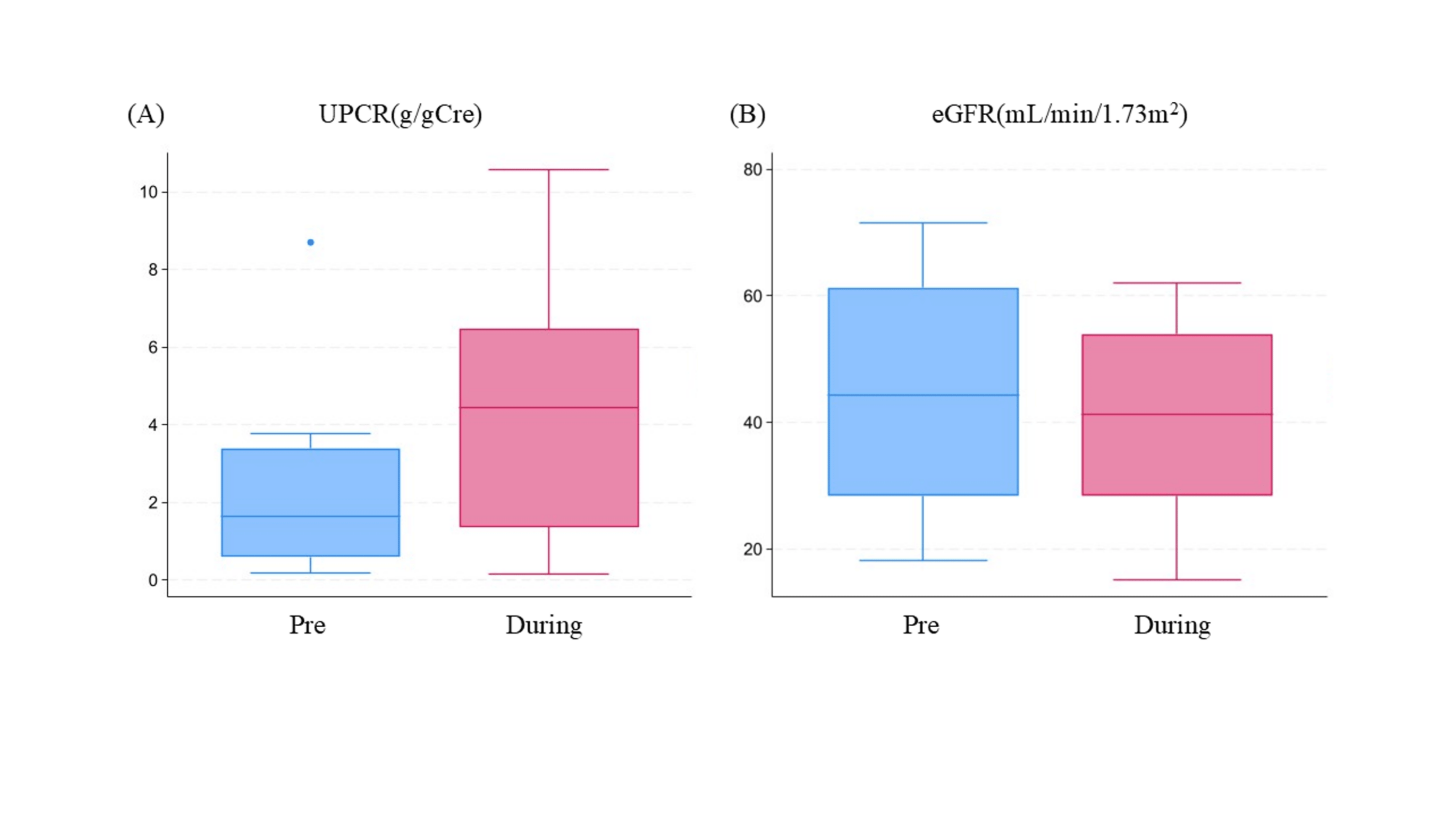
Box plots showing kidney functions before and during the COVID-19 epidemic (A) UPCR (g/gCre) and (B) eGFR (mL/min/1.73 m²). Box limits indicate 25 and 75 percentiles of the data, with a central line marking the median value. Lines extend from each box to capture the range of the remaining data, with dots placed past the line edges to indicate outliers. eGFR: estimated glomerular filtration rate; UPCR: urinary protein-to-creatinine ratio

Figure 2 shows a box-and-whisker diagram comparing renal function before and during COVID-19. eGFR during COVID-19 averaged 40.6 mL/min/1.73 m² (SD 16.5), and UPCR was 4.4 g/gCre (SD 3.45). The difference was -4.1 (SD 4.5) for eGFR and 1.9 (SD 5.2) for UPCR, with corresponding t-test p-values of 0.98 and 0.17, respectively.

Urine storage tests were performed in three of the eight cases where measurements were taken before and after the infection outbreak. The creatinine measurements by urine storage changed from 0.82 g/day to 0.89 g/day in Case 1, from 0.61 g to 0.49 g in Case 2, and from 1.40 g/day to 1.02 g/day in Case 3, respectively.

## Discussion

This study aimed to (1) compare the IPAQ short form with accelerometer-measured PA levels before the COVID-19 pandemic and (2) examine changes in PA and kidney function before and during the pandemic. The results showed that IPAQ tended to underestimate PA at all intensity levels compared to accelerometer data, especially at MVPA, which was statistically significant. Additionally, MVPA tended to decrease during the pandemic, while eGFR declined and urinary protein levels increased; however, none of these changes were statistically significant.

Few studies have compared IPAQ with accelerometer data in patients with NS. While validation studies of IPAQ and other PA questionnaires in different disease conditions exist, they generally report only moderate correlations, with a tendency to underestimate PA levels [7-9]. Our findings are consistent with those of previous studies. However, due to the small sample size, our study did not allow for stratified validation by age or sex. As noted earlier, questionnaire-based assessment of PA is inexpensive and easy to administer, but it is prone to recall bias and other inaccuracies, including potential underestimation, as demonstrated in the present study. In contrast, accelerometer-based measurements provide more accurate estimates of activity levels but are costly and time-consuming. While questionnaires remain a practical tool for large-scale studies, their use may introduce bias and should be interpreted with caution.

During the so-called first wave of the COVID-19 pandemic in Japan between April and June 2020, the government implemented a state of emergency to mitigate the spread of the virus [14-16]. The declaration was initially issued on April 7 for seven prefectures, including Tokyo, and was expanded nationwide on April 16. Under the emergency measures, residents were requested to refrain from nonessential outings, schools were closed, and restrictions were placed on business operations, including reduced hours for restaurants. These interventions led to significant changes in daily life and social activity. The number of newly confirmed cases peaked in mid-April and began to decline in May, prompting the full lifting of the state of emergency on May 25. During this period, approximately 17,000 cases and 900 deaths were reported nationwide [14]. These public health measures are believed to have had considerable effects on citizens’ behavior patterns, mental health, and PA. Studies examining PA changes due to the COVID-19 pandemic have reported inconsistent findings, likely due to differences in populations and measurement methods. In a study of Japanese workers, LPA and step count decreased, SB increased, and no significant difference was observed in MVPA [17]. Conversely, in a cohort of older Brazilian hypertensive patients (mean age 65.6 ± 3.8 years), LPA remained unchanged, while MVPA and step count tended to decline [18]. Our results showed similar trends to those of older patients with hypertension. A possible reason is that patients are a vulnerable population to treatment with antihypertensive drugs and immunosuppressive agents [1,13].

Although no statistically significant changes were observed, eGFR showed a stable to declining trend, while urinary protein levels tended to increase. Since eGFR is derived from serum creatinine, a potential confounding factor is muscle mass change. However, if a decrease in MVPA and step count led to a reduction in muscle mass, an increase in eGFR would be expected. Yet, during this period, the changes observed are in the opposite direction. For reference, three of the eight cases had creatinine measured in the stored urine, with two cases decreasing and one case increasing. Muscle mass may have tended to decrease, although there is no confirmation of the representativeness of these data. Notably, the observed increase in urinary protein levels warrants attention. Many chronic kidney disease (CKD) patients are concerned about the safety of exercise, as high-intensity exercise can induce transient proteinuria [19]. Studies have shown that strenuous exercise increases sympathetic nervous system activity and plasma catecholamine levels, enhancing glomerular capillary permeability and leading to proteinuria [11]. However, proteinuria typically returns to baseline within two hours post-exercise [12]. Recent randomized controlled trials (RCTs) have reported that exercise does not exacerbate proteinuria [20]. These RCTs included relatively few patients with nephrotic-range proteinuria, and the effects of changes in PA or rest in this patient group remain unclear. Our findings suggest that the impact of PA and rest in patients with NS may differ from that in other CKD populations.

A key strength of this study is that it is one of the few investigations examining the relationship between IPAQ and accelerometer-measured PA in patients with primary NS in the chronic phase. Additionally, it simultaneously assessed changes in both PA and kidney function. Another strength is that the study leveraged an unintended natural experiment in which the COVID-19 pandemic led to decreased PA. The changes observed before and during the pandemic are unlikely to have been influenced by factors other than the gradual reduction of treatment medications and COVID-19-related stress. However, the study is limited by its small sample size, precluding a comprehensive analysis of factors influencing kidney function, including adjusting for confounding factors and detecting statistical significance. Although this study shows a correlation between PA and kidney function, it does not determine whether PA independently affects kidney function, as potential confounding factors - such as diet, medication adherence, stress, inflammation levels, and socioeconomic status - were not controlled for. Additionally, as a single-center study, its findings may not be widely applicable. The degree of behavioral restrictions imposed by governments and other authorities also varies across countries and regions, further limiting the generalizability of the results. If the observed increase in urinary protein is accurate, potential causes, aside from changes in activity levels, could include the planned tapering of treatment medications, stress related to infectious disease outbreaks, and changes in medication adherence. Larger-scale studies are needed to address these issues.

## Conclusions

This study compared the IPAQ short form with accelerometer-measured PA and analyzed changes in activity and kidney function before and during the COVID-19 pandemic. Comparing IPAQ and accelerometer assessments, patients with NS tended to underestimate their activity in IPAQ assessments. Additionally, compared to the pre-COVID-19 epidemic period, MVPA and step count decreased during the epidemic, while SB and LPA tended to increase.

Given the lack of established guidelines for PA in patients with NS, further research is necessary to accumulate evidence and inform clinical recommendations.

## References

[1] (2021). KDIGO 2021 clinical practice guideline for the management of glomerular diseases. Kidney Int.

[2] Murase N, Katsumura T, Ueda C, Inoue S, Shimomitsu T (2002). Validity and reliability of Japanese version of International Physical Activity Questionnaire. J Health Welf Stat.

[3] Craig CL, Marshall AL, Sjöström M (2003). International physical activity questionnaire: 12-country reliability and validity. Med Sci Sports Exerc.

[4] Sallis JF, Saelens BE (2000). Assessment of physical activity by self-report: status, limitations, and future directions. Res Q Exerc Sport.

[5] Ekelund U, Tarp J, Steene-Johannessen J (2019). Dose-response associations between accelerometry measured physical activity and sedentary time and all cause mortality: systematic review and harmonised meta-analysis. BMJ.

[6] Jakicic JM, Kraus WE, Powell KE (2019). Association between bout duration of physical activity and health: systematic review. Med Sci Sports Exerc.

[7] Lee PH, Macfarlane DJ, Lam TH, Stewart SM (2011). Validity of the International Physical Activity Questionnaire Short Form (IPAQ-SF): a systematic review. Int J Behav Nutr Phys Act.

[8] Skender S, Ose J, Chang-Claude J (2016). Accelerometry and physical activity questionnaires - a systematic review. BMC Public Health.

[9] Rosa CS, Gracia-Marco L, Barker AR, Freitas IF Jr, Monteiro HL (2015). Assessment of physical activity by accelerometer and IPAQ-short version in patients with chronic kidney disease undergoing hemodialysis. Blood Purif.

[10] Okamoto S (2022). State of emergency and human mobility during the COVID-19 pandemic in Japan. J Transp Health.

[11] Poortmans JR, Haggenmacher C, Vanderstraeten J (2001). Postexercise proteinuria in humans and its adrenergic component. J Sports Med Phys Fitness.

[12] Fuiano G, Mancuso D, Cianfrone P (2004). Can young adult patients with proteinuric IgA nephropathy perform physical exercise?. Am J Kidney Dis.

[13] Wada T, Ishimoto T, Nakaya I (2021). A digest of the evidence-based clinical practice guideline for nephrotic syndrome 2020. Clin Exp Nephrol.

[14] (2025). Updates on COVID-19 in Japan. https://www.mhlw.go.jp/stf/seisakunitsuite/bunya/newpage_00032.html.

[15] Shimizu K, Wharton G, Sakamoto H, Mossialos E (2020). Resurgence of covid-19 in Japan. BMJ.

[16] Watanabe T, Yabu T (2021). Japan's voluntary lockdown. PLoS ONE.

[17] Fujii Y, Kitano N, Kai Y, Jindo T, Arao T (2024). Changes in accelerometer-measured physical activity and sedentary behavior from before to after COVID-19 outbreak in workers. J Epidemiol.

[18] Browne RA, Macêdo GA, Cabral LL (2020). Initial impact of the COVID-19 pandemic on physical activity and sedentary behavior in hypertensive older adults: an accelerometer-based analysis. Exp Gerontol.

[19] Bellinghieri G, Savica V, Santoro D (2008). Renal alterations during exercise. J Ren Nutr.

[20] Yang L, Wu X, Wang Y, Wang C, Hu R, Wu Y (2020). Effects of exercise training on proteinuria in adult patients with chronic kidney disease: a systematic review and meta-analysis. BMC Nephrol.

